# Extract of *Pfaffia glomerata* Ameliorates Paroxetine-Induced Sexual Dysfunction in Male Mice and the Characterization of Its Phytoconstituents by UPLC-MS

**DOI:** 10.3390/foods12173236

**Published:** 2023-08-28

**Authors:** Qianqian Huang, Haiying Wu, Xiaoming Qin

**Affiliations:** 1 Guangdong Provincial Key Laboratory of Aquatic Products Processing and Safety, Guangdong Provincial Science and Technology Innovation Center for Subtropical Fruit and Vegetable Processing, College of Food Science and Technology, Guangdong Ocean University, Zhanjiang 524088, China; 2112103094@stu.gdou.edu.cn (Q.H.); 2112103049@stu.gdou.edu.cn (H.W.); 2 National Research and Development Branch Center for Shellfish Processing, Zhanjiang 524088, China

**Keywords:** *Pfaffia glomerata*, extract, sexual function, molecular docking, paroxetine

## Abstract

*Pfaffia glomerata* extract (PGE) has a variety of biological activities. However, its ameliorative effect on and exact working mechanism in male sexual dysfunction are still poorly understood. This study aims to evaluate the ameliorative effect of PGE on paroxetine (PRX)-induced sexual dysfunction in male mice and uses molecular docking technology to investigate its underlying mechanism. In this work, PRX-induced sexual dysfunction was caused and PGE was gavaged in mice for 28 days. The results show that PGE significantly improved the sexual performance of mice and reduced the damage to testicular tissues. Further studies showed that PGE restored serum sex hormones to normal levels and increased nitric oxide (NO) and cyclic guanosine monophosphate (cGMP) levels as well as nitric oxide synthase (NOS) activity in penile tissues, while also decreasing phosphodiesterase-5 (PDE-5) activity, thereby maintaining normal penile erection in mice. In addition, PGE improved the activities of enzymes (LDH, ACP, and ALP) related to energy metabolism in the testis and significantly increased sperm count and viability in mice. Furthermore, the molecular docking results show that all eight compounds in PGE could form a stable complex with PDE-5 and inhibit the activity of PDE-5. In conclusion, PGE had an ameliorative effect on PRX-induced sexual dysfunction, suggesting that PGE has a potential protective effect on male sexual health.

## 1. Introduction

Male sexual dysfunction is a disease caused by many factors, such as living habits, chronic diseases, and drug side effects [1]. It is usually manifested as erectile dysfunction (ED), abnormal libido, abnormal ejaculation, and pain during sexual intercourse [2]. ED entails the penis not being able to achieve or maintain sufficient erectile hardness to complete a satisfactory sexual life [3]. Disorders of sex hormone levels, decreases in nitric oxide (NO) and cyclic guanosine monophosphate (cGMP) levels, and an increase in 5-phosphodiesterase (PDE5) activity in the cavernous body of the penis are the main causes of ED [4]. According to statistics, more than 150 million men worldwide are affected by ED, and it is estimated that the number of male ED patients will increase to 322 million by 2025 [5]. Sexual dysfunction caused by drug side effects is an important reason. For example, selective serotonin reuptake inhibitors (SSRIs) are the first-line drugs for the treatment of depression, which have obvious side effects on male sexual ability. Paroxetine (PRX) is a short-acting, highly selective SSRI. Many studies have shown that the continuous use of PRX can lead to ED, decreased libido, and abnormal ejaculation [6]. Therefore, PRX is usually used as a modeling drug to establish a sexual dysfunction model of rodents to evaluate the improvement in sexual dysfunction by plants [7,8]. However, the commonly used drug PDE5 inhibitor for the treatment of sexual dysfunction, sildenafil (SDF), may cause a series of side effects, such as headache, abdominal pain, vomiting, facial congestion, and indigestion [9]. Therefore, it is necessary to use natural active substances that have non-toxic side effects to create safe and effective natural products to prevent and improve male sexual dysfunction. Studies have reported that many plant extracts contain saponins, flavonoids, phenolics, steroids, alkaloids, triterpenes, and other active ingredients, which can be used as PDE5 inhibitors and have a strong effect on improving male sexual function [10].

*Pfaffia glomerata*, Amaranthaceae, of the *Faffia* genus, is a perennial herb [11]. It is native to the Amazon River Basin in South America, encountered in countries such as Brazil, Panama, Peru, and other tropical rainforest areas [12]. It has also been successfully planted and cultivated in Guangdong, Guangxi, Zhejiang, and other places in China [13]. *Pfaffia glomerata* root is rich in pfameric acid, saponins, oleanolic acid, and other triterpenoids [14]. Previous studies have shown that *Pfaffia glomerata* has anti-inflammatory [15], anti-cancer and antibacterial [16], and antioxidant [17] activities. *Pfaffia glomerata* is also regarded as a functional component to promote health benefits because of its biological activity; in 1994, the US FDA approved it as a healthy food item [18]. Some *Pfaffia glomerata* powder and its extract products are even used as nutritional supplements for athletes to promote athletes’ endurance and help the body to adapt to external pressures [19]. In addition, *Pfaffia glomerata* also has the effect of improving sexual dysfunction [20], but the research is not systematic and comprehensive enough, the relevant reports are still scarce, and the theoretical basis is relatively insufficient; therefore, more comprehensive scientific research is still needed.

Therefore, this study aims to investigate the ameliorative effects of PGE on PRX-induced sexual behavior, testicular histopathology, penile-erection-related indexes, testicular marker enzyme activities, and sperm parameters in male mice. In addition, using ultra-high performance liquid chromatography time-of-flight mass spectrometry (UPLC-XEVO G2 XS QTQF) and computer molecular docking technology, the inhibitory effects of the active ingredients in PGE on PDE-5 are evaluated, and its mechanism of action is preliminarily explored.

## 2. Materials and Methods

### 2.1. Plant Material

The *Pfaffia glomerata* (artificial planting for less than 5 years; it is in the 3rd to 4th year of seedling flowering period) experimental materials used in this study were collected from the under-forest economic and technological innovation center of Leizhou Peninsula in Guangdong Province. Professor Suqing Liu of the Binhai Agricultural College of Guangdong Ocean University introduced and planted it from the Guangxi Botanical Garden of Medicinal Plants. The experimental materials were identified as *Pfaffia glomerata* by Professor Liying Yu (Tel.: +86-13006915609) from the Guangxi Botanical Garden of Medicinal Plants and The Institute of Medicinal Plant Development (IMPLAD), according to plant identification methods.

### 2.2. Experimental Drugs

Paroxetine Hydrochloride tablets (H10950043) were purchased from Sino-US Tianjin Shike Pharmaceutical Co., Ltd. (Tianjin, China). Sildenafil Citrate tablets (H20020527) was purchased from Pfizer Pharmaceutical Co., Ltd. (Dalian, China). The oleanolic acid standard were purchased from McLean Biotechnology Co., Ltd. (Shanghai, China). Chikusetsusaponin IVA was purchased from Shanghai Yuanye Biotechnology Co., Ltd. (Shanghai, China). 20Hydroxyecdysone was purchased from Aladdin Industrial Corporation (Shanghai, China). Carboxymethyl cellulose sodium was purchased Beijing Kulebo Technology Co., Ltd. (Beijing, China). All chemicals and reagents used were of analytical grade and are commercially available.

### 2.3. Preparation of Pfaffia glomerata Extract (PGE)

The fresh *Pfaffia glomerata* (underground roots were the raw materials) was washed, sliced, vacuum freeze-dried (FD-551, Tokyo, Japan), and crushed through a 60-mesh sieve. PGE were prepared from the *Pfaffia glomerata* powder according to the method of Fu et al. [21] and the method of Luo [22]. Briefly, 100 g of *Pfaffia glomerata* powder were mixed with 3000 mL of 70% ethanol (1: 30 *w*/*v*). The mixture was extracted at 350 w at 60 °C for 1 h by using a ultrasonic instrument (KQ-500DB, Kunshan, China). Subsequently, the extract was cooled and centrifuged (Lynx6000, Thermo, Waltham, MA, USA) at 8000 r/min at 4 °C for 15 min to obtain the supernatant. It was extracted twice under the same conditions, and the two extracts were combined. The samples were concentrated (N-1300, EYELA, Tokyo, Japan) and freeze-dried (FD-551, Tokyo, Japan) into a powder, stored at −20 °C in the dark for later use.

### 2.4. Determination of Saponin Content

The content of saponins in the PGE was determined referring to Wei’s et al. [23] method at a wavelength of 545 nm, with slight modifications. First of all, 0.3 g PGE powder were mixed with 10 mL of 2% H_2_SO_4_; the mixture was hydrolyzed by acid at 350 w at 60 °C for 1.5 h by using an ultrasonic instrument (KQ-500DB, Kunshan, China). The acid hydrolysate was cooled, diluted, and extracted with ethyl acetate 4 times, 20 mL/time. The ethyl acetate extract was combined and concentrated to obtain triterpenoid sapogenin (oleanolic acid) extract. The extract was placed in a 50 mL volumetric flask and diluted with methanol to scale. Secondly, 0.2 mg of the oleanolic acid standard was dissolved in methanol and diluted to 10 mL volumetric flask to obtain 0.2 m/mL standard solution. Then, 0.2 mL of the standard solution and sample solution were taken in the test tube and evaporated to dryness in a hot water bath. Subsequently, 0.2 mL of newly prepared 5% vanillin-glacial vinegar and 0.8 mL of perchloric acid were added to the test tube, and the color was developed in a water bath at 70 °C for 15 min and cooled. Finally, 5 mL of glacial acetic acid were added and shaken well; 200 μL samples from each test tube were inserted in 96-well plates, and the absorbance value was measured with a microplate reader (Varioskan Flash, Thermo, Waltham, USA) at a wavelength of 545 nm. This procedure was repeated three times.

The calculation formula is:

$$X=\frac{Y\times V_{1}\times A}{V_{2}}$$

where *X*—oleanolic acid content, mg/g; *Y*—oleanolic acid mass measured by standard curve, mg; *A*—sample dilution multiple; *V*_1_—total volume of the sample, mL; and *V*_2_—determination of the sample volume, mL.

*Pfaffia glomerata* saponin content (%) = oleanolic acid content × 1.64 (Reduction coefficient) [24,25].

### 2.5. PGE Characterization by UPLC–MS

UPLC-MS has been widely used in the qualitative and quantitative analyses of various plant bioactive substances [26,27]. The triterpenoids in PGE were qualitatively analyzed referring to Mroczek’s et al. [28] method by ultra-high performance liquid chromatography time-of-flight mass spectrometry (UPLC-XEVO G2 XS QTQF, Waters, MA, USA). In this work, the chromatographic column used was Waters-C18 column (2.1 × 50 mm, 1.7 μm), and the mobile phases used were 0.1% formic acid water (A) and 0.1% formic acid acetonitrile (B). The sample (1 μL) was first eluted for 0~0.5 min with 70% mobile phase (A), and eluted for 0.5~8.1 min from 70%~30% mobile phase (A), and then eluted for 8.1~10 min with 30% mobile phase (A); it was subsequently eluted for 10~10.5 min from 30~70% mobile phase (A), and finally eluted for 10.5~12 min with 70% mobile phase (A). The flow rate was 0.2 mL/min, and the injection concentration was 5 μg/mL. After chromatography, the ESI-MS/MS analysis was conducted in the negative ion mode. The specific parameters were set as: collision energy: 6 eV; capillary voltage: 1.8 kV; cone hole gas flow: 50 L/h; desolvation gas flow rate: 700 L/h; and source temperature, 120 °C. The sample was scanned in the range of 150–1500 m/z to obtain the total ion current diagram.

### 2.6. Animals and Treatment

**Figure 1:**
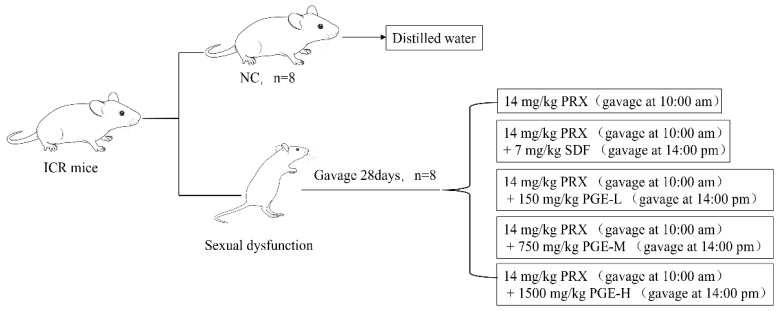
Animals and treatment.

In this study, all experimental animal protocols and procedures were approved by The Laboratory Animal Committee of Guangdong Ocean University (China (GDOU-LAE-2022-032)). A total of 96 healthy SPF ICR mice (26 ± 2 g) (Animal license number SCXK (Beijing) 2019-0010, half male and half female, were purchased from Guangzhou Yancheng Biotechnology Co., Ltd. (Beijing, China). All mice were raised in a sterile environment, temperature of 22~26 °C, humidity of 50%~60%, and alternating light and dark conditions for 12 h (10:00 a.m. to 22:00 p.m.), with free access to feeding (C60 irradiation test for mice-maintained feed) and drinking water. After seven days of acclimatization, all male mice were randomly divided into 6 groups (*n* = 8). The grouping details are shown in Figure 1: control group (CN, equal volume of distilled water), PRX group (14 mg/kg PRX (calculated as C_19_H_20_FNO_3_)), PRX + SDF group (14 mg/kg PRX + 7 mg/kg SDF (calculated as C_22_H_30_N_6_O_4_S·C_6_H_8_O_7_)), PRX + PGE low-dose group (14 mg/kg PRX + 150 mg/kg PGE-L), PRX + PGE medium-dose group (14 mg/kg PRX + 750 mg/kg PGE-M), and PRX + PGE high-dose group (14 mg/kg PRX + 1500 mg/kg PGE-H). The gavage volume of mice was 0.1 mL/10 g. The dose of PRX and SDF was based on the study of Ayokunle O et al. [29] and was suspended in 1% carboxymethyl cellulose sodium. The dose of PGE was based on the general recommended dose of ginseng in ‘Chinese Pharmacopoeia’. Among them, the PGE-L, PGE-M, and PGE-H dose groups were designed according to the general recommended dose of adults and 5 times and 10 times of the general recommended doses. All mice in the experimental groups were administered oral gavage for 28 days. The PRX, SDF, and PGE were gavaged separately. The PRX was gavaged orally at 10:00 in the morning, and SDF and PGE were gavaged orally at 14:00 in the afternoon separately.

### 2.7. Sexual Behavior Experiment

Sexual behavior experiments were performed 60 min after the end of the last oral gavage. Forty-eight hours before formal sexual behavior female mice were intramuscularly injected with 0.2 mg/mouse estradiol benzoate injection, and 1 mg/kg progesterone injection was injected in the first 4 h to make female mice in estrus [1]. The sexual experiment was conducted at 20:00–23:00 p.m. in a quiet room with a dark red light. Male mice were first placed in a clear plastic box (30 × 15 × 15 cm) for 15 min and recorded by a high-definition camera. The sexual behavior parameters of the first 30 min were observed and recorded from the time when female mice were placed in the box. We recorded the mount latency (ML, time from the introduction of the female until the first mount (no vaginal penetration)), mount frequency (MF, the number of mounts in 30 min), intromission latency (IL, the time of the first intromission of male mice into the vagina of female mice), intromission frequency (IF, total number of introductions from female to male at the end of the experiment), ejaculation latency (EL, the time interval from the first introduction to ejaculation in male mice), and post-ejaculation interval (PEL, time interval from ejaculation to reintroduction in male mice) [7].

### 2.8. Organ Coefficient

At 10:00 on the next day after the sexual behavior test, the mice were weighed and sacrificed by enucleation of the eye and bled for the collection of blood [30]. The penis, testis, heart, thymus, lien, liver, and ren of the mice were taken out and accurately weighed using an electronic balance (OHAUS, Hangzhou, China) to calculate the organ coefficient (organ weight/body weight).

### 2.9. Measurements of Hormones and Enzymes

The measurements of hormones and enzymes were conducted referring to Zhang’s et al. [31] method for determination and analysis. We determined mouse serum sex hormone levels, including testosterone (T), luteinizing hormone (LH), follicle-stimulating hormone (FSH), and estradiol (E2). According to the instructions of the enzyme-linked immunosorbent assay (ELISA) kit (Yancheng China), the blood of the mice was collected. After natural coagulation for 15 min, the blood was centrifuged at 3000 r/min at 4 °C for 15 min (UNIVER 320 R, Tutlingen, Germany), and the serum was collected. The penile tissue homogenates were prepared according to the instructions of the kit. The content of nitric oxide (NO) and the activity of nitric oxide synthase (NOS) in the penile tissue homogenate were determined by biochemical kit (Nanjing Jiancheng Institute of Bioengineering, Nanjing, China). The content of cyclic guanosine monophosphate (cGMP) and the activity of phosphodiesterase-5 (PDE-5) in the penile homogenate were determined by ELISA kit (ELISA, Yancheng, China). The right testicular homogenate of mice was prepared according to the kit instructions, and the acid phosphatase (ACP) activity, alkaline phosphatase (ALP) activity, lactate dehydrogenase (LDH) activity, and malondialdehyde (MDA) content in the homogenate were measured (Nanjing Jiancheng Bioengineering Institute, Nanjing, China). The protein concentrations of the penile and testicular tissue homogenates were measured by (BCA) protein assay kit (Beyotime Biotechnology, Shanghai, China). All samples were stored in a refrigerator (HFLTP 86(580), Hefei, China) at −80 °C before analysis.

### 2.10. Testis Histopathological and Sperm Analysis

The left testis of mice was fixed with formaldehyde fixative, embedded, sliced, and stained with hematoxylin and eosin (H&E), and the images were collected. The left and right epididymis of the mice were cut into pieces in an EP tube containing normal saline, and then shaken and incubated in a water bath at 37 °C for 15 min [32]. The sperm quality was analyzed according to the method of Oghbaei et al. [33]. A drop of diluted diluent was taken on the blood cell count, and the number of sperm in five squares was counted under a biological microscope (CX33, Olympus Corporation, Tokyo, Japan). The number of sperm per milliliter was equal to N × dilution factor × 5 × 10^4^. Another drop of diluent was taken to smear; 200 sperms were observed, and the number of active sperm was counted. In addition, the sperm was stained according to the instructions of the quick sperm stain kit (Nanjing Jiancheng Bioengineering Institute, Nanjing, China), and a fluorescence-inverted microscope–spectrometer coupling (DMI4000B+Iso Plane 160, Leica Corporation, Wetzlar, Germany) was used to observe and capture the stained sperm images.

$$Sperm\;motility=\frac{Active\;sperncount}{200}\times 100\%$$

### 2.11. Molecular Docking

ChemDraw20.0 and PDB database (https://www.rcsb.org/ (accessed on 10 June 2023)) were used to download the related active ingredients and PDE-5 structure (PDB DOI: https://doi.org/10.2210/pdb2H42/pdb (accessed on 10 June 2023)) [34], respectively. The active ingredients were treated by adding atomic charges through the AutoDock Tools 1.5.6 software; the excess chain and water of the receptor protein were removed and hydrogenated, etc. [35]. The molecular docking analysis was conducted referring to the research of Khalid et al. [1]. The coordinates of the active site were determined by referring to the binding site of SDF in the crystal structure (39.50, 119.11, and 12.62). Then, AutoDock Vina was used to perform molecular docking along the coordinate axis (90, 108, and 80). The binding energy ≦ −5 kcal/moL indicated that the docking result was good. Finally, Pymol 2.6 software was used to visualize the three-dimensional structure of the docking result, and Discovery Studio 2019 was used to visualize the two-dimensional structure.

### 2.12. Statistical Analysis

All data were analyzed by SPSS 27, Excel 2021, GraphPad 9.3 software for statistical analysis and drawing. Data were analyzed by a one-way analysis of variance. *p* < 0.05 indicated that the difference was significant, *p* < 0.01 indicated that the difference was very significant, and *p* < 0.001 indicated that the difference was extremely significant; all three were statistically significant. All experimental results are expressed as mean ± standard deviation (X ± SD).

## 3. Results

### 3.1. Analysis of the Saponin Content of PGE

After extraction with 70% ethanol, the content of triterpenoid saponins in PGE was 12.28%. Triterpenoid saponins are one of the main active substances of *Pfaffia glomerata* [36]. In previous studies, Liu et al. [37] found that it has anti-fatigue effect, and Oshima et al. [38] found that it can promote the synthesis and secretion of sex hormones. In this study, the saponin content in PGE was 12.28%, which could be preliminarily judged to have the potential to improve male sexual dysfunction.

### 3.2. UPLC–MS Analysis

**Figure 2:**
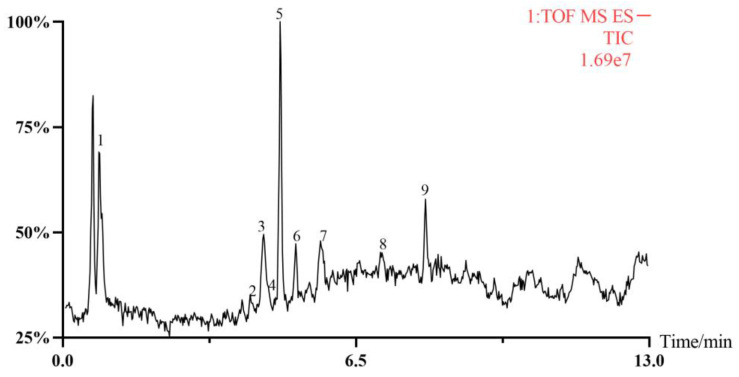
Total ion current diagram (The numbers in the Figure 2 correspond to the compounds with numbers in Table 1, respectively).

According to the conditions described in Section 2.5, the PGE sample was analyzed by HPLC-MS, and the total ion flow diagram is shown in Figure 2. Through reference and comparison with the standard substances, nine main compounds were inferred from PGE, namely: 20-Hydroxyecdysone (1), Sulfachyranthoside B (2), 3-O-β-D-Glc-(1→2)-[[2-carboxy-1-(carboxymethyl)-2-hydroxyethyl-(1→3)]-β-D-GluA oleanolic acid (3), Achyranthoside E (4), Chikusetsusaponin IVA (5), Pfaffiaglycoside B (6), Zingibroside R1 (7), Achyranthoside C (8), and Calenduloside E (9); see Table 1.

### 3.3. Effect of PGE on the Sexual Behavior of Male Mice

**Figure 3:**
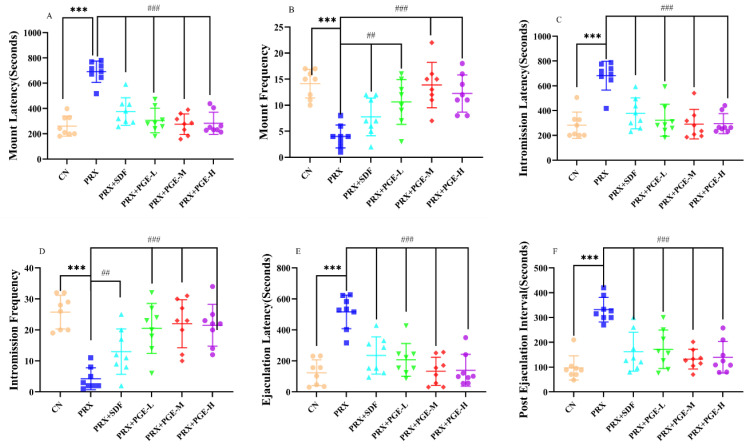
Effect of PGE on the sexual behavior of male mice ((**A**) ML; (**B**) MF; (**C**) IL; (**D**) IF; (**E**) EL; and (**F**) PEL). The data are expressed as mean ± SD, *n* = 8. Compared with the CN group, *** indicated extremely significant difference (*p* < 0.001). Compared with the PRX group, ## means very significant difference (*p* < 0.01), and ### means extremely significant difference (*p* < 0.001).

The sexual ability of mice can be reflected by sexual behavior indicators. From Figure 3, it can be seen that, compared with the CN group, the mount latency, introduction latency, ejaculation latency, and post-ejaculation interval of the PRX group were significantly prolonged (*p* < 0.001), and the mount frequency and introduction frequency were significantly reduced (*p* < 0.001). However, compared with the PRX group, the PRX + SDF group and the PRX + PGE groups had mount latency, introduction latency, and ejaculation latency that were significantly shortened (*p* < 0.001 and *p* < 0.01, respectively), and the mount frequency increased significantly (*p* < 0.001 and *p* < 0.01, respectively). In addition, compared with the PRX group, the introduction frequency of the PRX + SDF group and the PRX + PGE group was significantly increased (*p* < 0.001; *p* < 0.01, respectively), and the post-ejaculation interval was significantly shortened (*p* < 0.001). The results of the sexual behavior analysis show that paroxetine caused a decrease in the sexual ability of mice, while SDF and PGE significantly improved the sexual ability of mice.

### 3.4. Effect of PGE on Organ Coefficient

It can be seen from Table 2 that there was no significant difference in visceral coefficient and penis coefficient among the PRX group, PRX + SDF group, and PRX + PGE group compared with the CN group (*p* > 0.05). Compared with the CN group, the testicular coefficient of the PRX group decreased significantly (*p* < 0.01). However, compared with the PRX group, the testicular coefficient of the PRX + SDF group, the PRX + PGE-L group, the PRX + PGE-M group, and the PRX + PGE-H group increased significantly (*p* < 0.01, *p* < 0.05), by 9.0%, 9.2%, 8.9%, 12.3%, and 11.8%, respectively. It can be preliminarily judged from the results of the testicular coefficient that PGE can reduce the damage of paroxetine to mouse testis and has the potential to improve sexual function.

### 3.5. Effects of PGE on Serum Sex Hormones

**Figure 4:**
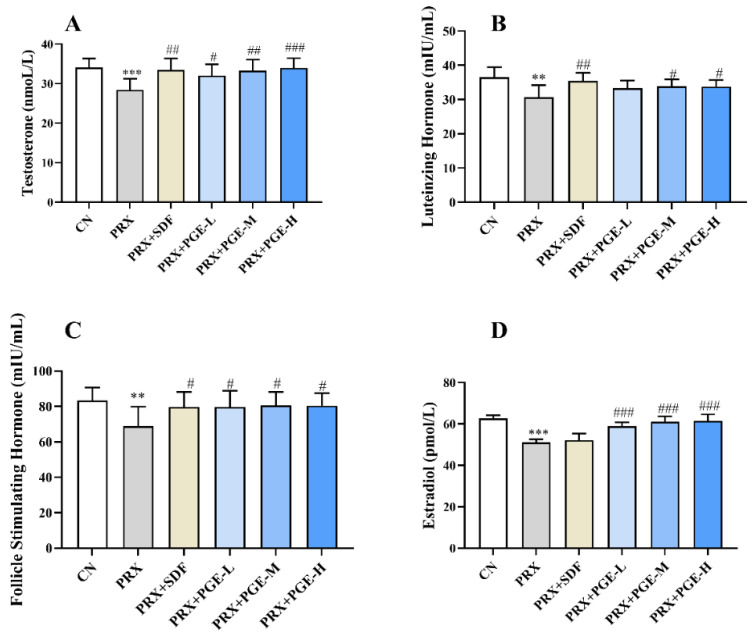
Effect of PGE on serum sex hormones ((**A**) T; (**B**) LH; (**C**) FSH; and (**D**) E2). The data are expressed as mean ± SD, *n* = 8. Compared with the CN group, ** indicates very significant difference (*p* < 0.01), and *** indicates extremely significant difference (*p* < 0.001). Compared with the PRX group, # means a significant difference (*p* < 0.05), ## means very significant difference (*p* < 0.01), and ### means extremely significant difference (*p* < 0.001).

Sex hormones can stimulate sexual maturity and are indispensable in sexual behavior. The levels of T, LH, FSH, and E2 in serum are indispensable indicators for evaluating the strength of sexual function. As shown in Figure 4, PRX significantly suppressed the concentration of serum sex hormones in mice, including T, LH, FSH, and E2 (*p* < 0.001, *p* < 0.01). Obviously, PRX interfered with the serum sex hormone levels of mice. Compared with the PRX group, different doses of PGE significantly increased the concentrations of T, FSH, and E2 (*p* < 0.01, *p* < 0.01, *p* < 0.05); the doses of PGE-M and PGE-H also significantly increased the concentration of LH (*p* > 0.05). Meanwhile, the SDF treatment also significantly increased the concentrations of T, LH, and FSH (*p* < 0.01, *p* < 0.05).

### 3.6. The Effect of PGE on the Activity or Content of NO, NOS, cGMP, and PDE5 in the Penile Tissue

**Figure 5:**
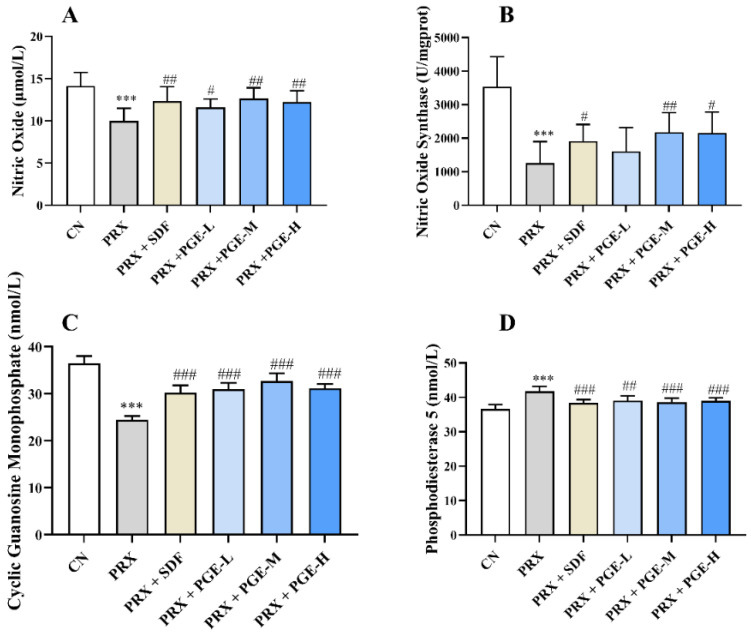
Effect of PGE on the contents of NO, NOS, cGMP, and PDE5 in the penile tissue ((**A**) NO; (**B**) NOS; (**C**) cGMP; and (**D**) PDE-5). The data are expressed as mean ± SD, *n* = 8. Compared with the CN group, and *** indicates extremely significant difference (*p* < 0.001). Compared with the PRX group, # means a significant difference (*p* < 0.05), ## means very significant difference (*p* < 0.01), and ### means extremely significant difference (*p* < 0.001).

NO content, NOS activity, cGMP content, and PDE5 activity in the penile tissue play an important role in male erectile function. As shown in Figure 5, the contents of NO and cGMP and the activity of NOS were significantly decreased in the PRX group, and the activity of PDE-5 was significantly increased with the CN group (*p* < 0.001). Compared with the PRX group, the treatment of PGE restored the contents of NO and cGMP (*p* < 0.001, *p* < 0.01, and *p* < 0.05) and the activity of NOS (*p* < 0.01, *p* < 0.05). In addition, compared with the PRX group, PGE significantly reduced the activity of PDE5 in the penile tissue (*p* < 0.001, *p* <0.01) and restored the activity of PDE5 to a level close to that of the CN group. At the same time, SDF, as a classic PDE-5 inhibitor, also restored PRX-induced erectile dysfunction.

### 3.7. The Effect of PGE on the Activity and Contents of LDH, ACP, ALP, and MDA in the Testicular Tissue

**Figure 6:**
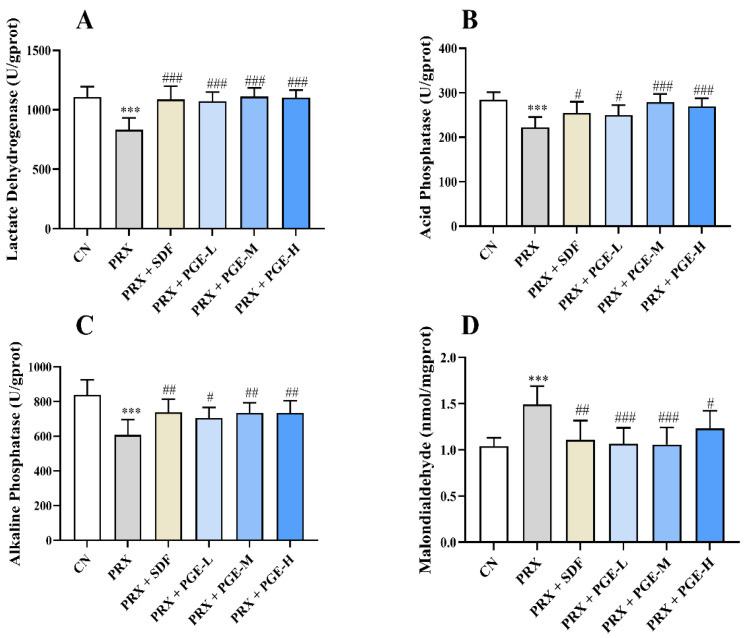
Effect of *Pfaffia glomerata* on the activity and contents of LDH, ACP, AKP, and MDA in the testis tissue ((**A**) LDH; (**B**) ACP; (**C**) AKP; and (**D**) MDA). The data are expressed as mean ± SD, *n* = 8. Compared with the CN group, and *** indicates extremely significant difference (*p* < 0.001). Compared with the PRX group, # means a significant difference (*p* < 0.05), ## means very significant difference (*p* < 0.01), and ### means extremely significant difference (*p* < 0.001).

LDH, ACP, and ALP are testicular marker enzymes that play an important role in spermatogenesis [43]. When the testicular tissue is damaged, its marker enzyme activity also changes accordingly, thus affecting the generation and quality of sperm. According to Figure 6, the activities of LDH, ACP, and ALP in the PRX group were significantly lower than those in the CN group (*p* < 0.001). However, the activities of LDH, ACP, and ALP in the PRX + SDF group and the PRX + PGE group were significantly increased to levels close to those of the CN group compared with the PRX group (*p* < 0.001, *p* < 0.01, and *p* < 0.05), and those of the PGE-M group were increased by 33.7%, 25.5%, and 20.9%, respectively. The results show that both SDF and PGE restored the decrease in testicular marker enzyme activity caused by PRX. MDA is an important antioxidant index, and from the results of Figure 6D, it can be seen that the MDA content in the testis tissue of PRX group is 1.4 times that of the CN group (*p* < 0.001), while the MDA content in the testis tissue of the PRX + SDF group, PRX + PGE-L group, PRX + PGE-M group, and PRX + PGE-H group was, respectively, reduced by 25.5%, 28.2%, 28.9%, and 17.4% (*p* < 0.001, *p* < 0.01, and *p* < 0.05). It can be seen that the PGE treatment is effective in reducing the PRX-induced MDA elevation in the testicular tissue.

### 3.8. Histopathological Analysis

**Figure 7:**
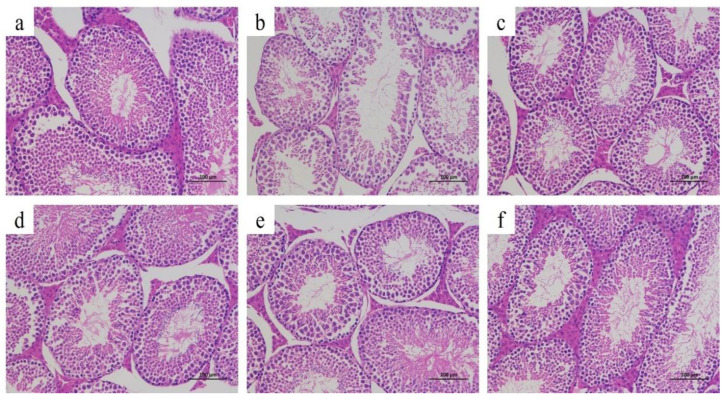
Pathological section analysis of penis and testis tissues: (**a**) represents the CN group, (**b**) represents the PRX group, (**c**) represents the PRX + SDF group, (**d**) represents the PRX + PGE-L group, (**e**) represents the PRX + PGE-M group, and (**f**) represents the PRX + PGE-H group.

According to the results of the H&E staining of testicular tissue in Figure 7, the seminiferous tubules of mice in the CN group were closely arranged, the number of spermatogenic cells was abundant, the arrangement was uniform and orderly, and the morphology was good. The number of spermatogenic cells in the PRX group was significantly reduced, the cell distribution was loose and disordered, and obvious vacuolization appeared in the lumen. Compared with the PRX group, the number of spermatogenic cells in the seminiferous tubules of the PRX + SDF group and PRX + PGE group was significantly increased; the cells were arranged neatly and the morphology was significantly improved. The results of HE staining of testicular tissue show that PGE can alleviate the testicular injury caused by PRX.

### 3.9. Effects of Pfaffia Glomerata on Sperm Count and Viability

**Figure 8:**
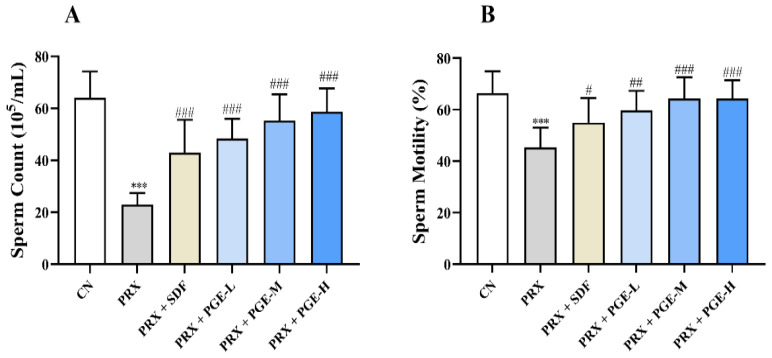
**(A): Sperm count; (B): Sperm motility.** The data are expressed as mean ± SD, *n* = 8. Compared with the CN group, and *** indicates extremely significant difference (*p* < 0.001). Compared with the PRX group, # means a significant difference (*p* < 0.05), ## means very significant difference *(p* < 0.01), and ### means extremely significant difference (*p* < 0.001).

**Figure 9:**
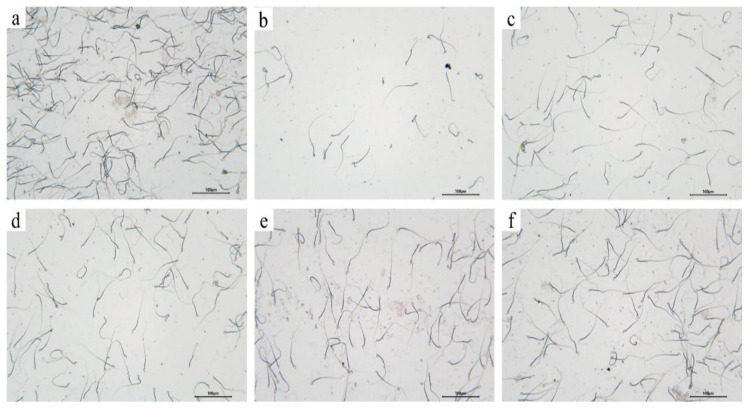
Sperm quality: (**a**) represents the CN group, (**b**) represents the PRX group, (**c**) represents the PRX + SDF group, (**d**) represents the PRX + PGE-L group, (**e**) represents the PRX + PGE-M group, and (**f**) represents the PRX + PGE-H group.

As shown in Figure 8, compared with the CN group, the sperm count and sperm motility of the PRX group were significantly decreased (*p* < 0.001), while PGE administration significantly improved the sperm count and sperm motility (*p* < 0.001, *p* < 0.01, and *p* < 0.05). In addition, as shown in Figure 9, PRX administration also led to abnormal sperm in male mice, such as coiled tails, curved tails, annular tails, headless, and double heads (see Figure 9b), consistent with the results of Saikia Q et al. [8]. It can be seen that PRX not only causes ED in male mice, but also has an adverse effect on sperm quality in mice. This result is consistent with the trend of the above results of the decreased enzyme activities of testicular marker enzymes (ACP, ALP, and LDH) affecting spermatogenesis and the results of the H&E-stained sections of the testicular tissue.

### 3.10. Molecular Docking Analysis

**Figure 10:**
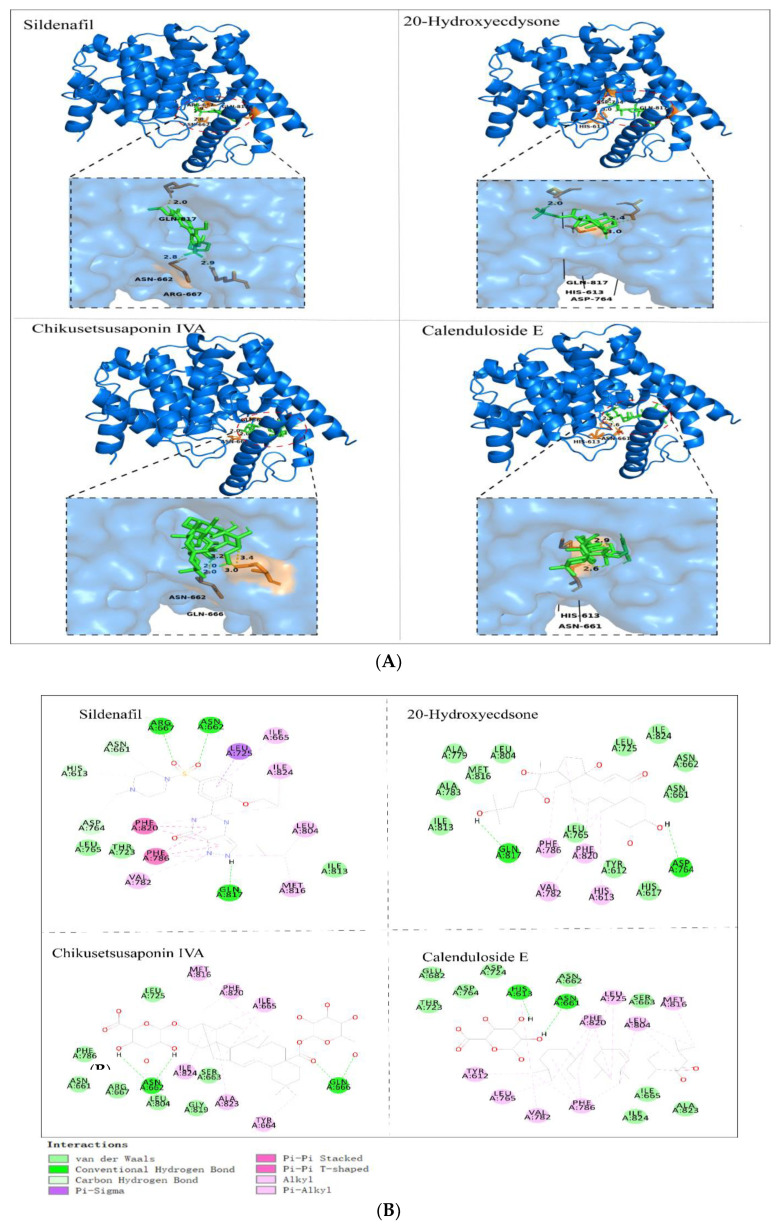
(**A**) Three-dimensional conformation of PDE-5 docked with different ligand compounds by AutoDock Vina. (**B**) Discovery studio 2019 shows the two-dimensional structure of the docking results.

Molecular docking is a technique in which small-molecule ligands are combined with macromolecular receptors through various interactions [44]. The positive drug SDF and the PGE component were paired with the active site of PDE-5, and both formed stable complexes with similar postures in the active pocket. Figure 10 shows the three-dimensional and two-dimensional maps of the complexes of the first three compounds with PDE5 in PGE with higher response values. The binding energy and force of the interaction between each compound and PDE-5 are shown in the following table (Table 3). These interactions are the main reasons for maintaining the stability of the complex [45].

## 4. Discussion

The continuous use of SSRIs, especially PRX, can lead to male sexual dysfunction, including ED, decreased libido, and ejaculation disorders [46]. A large number of studies have shown that the sexual behavior parameters of rodents change significantly after taking PRX, including the prolongation of ML, IL, and EL and the decrease in MF and IF. The changes in these sexual behavior parameters reflect the decrease in the sexual ability of male rats after taking PRX [6,47]. The index of sexual behavior is one of the most intuitive and important indexes to reflect the sexual ability of experimental animals. When one of the parameters is positive, it can be used to judge whether the sample has a certain effect on improving sexual dysfunction [48]. In this study, the PRX group significantly prolonged the ML, IL, EL, and PEL of male mice and significantly reduced the MF and IF, while the SDF and PGE treatments reversed these changes.

NO and cGMP are the key factors in the NO/cGMP penile erection pathway. After NO is diffused into smooth muscle cells, guanylate cyclase (GC) is activated. GC converts inactive guanosine triphosphate (GTP) into active cGMP. The increase in cGMP concentration can reduce the concentration of calcium ions in smooth muscle cells, thus causing smooth muscle relaxation and penile erection [49]. Therefore, maintaining the concentration of NO and cGMP in smooth muscle cells is crucial in penile erection. PDE-5 can degrade cGMP, which is the main reason for the decrease in cGMP concentration in smooth muscle cells. Therefore, inhibiting the activity of PDE-5 can increase the concentration of cGMP, thus maintaining penile erection [50]. It has been reported that taking PDE5 inhibitors before sexual intercourse is effective for most patients. For example, SDF can significantly improve erectile dysfunction and enhance penile stiffness in patients, but the drug also has some side effects in some patients, including headaches, abdominal pain, facial congestion, indigestion, and even damage to the retina [51]. Therefore, it is urgent to find natural active substances with non-toxic side effects to replace these drugs in the treatment of erectile dysfunction. Studies have found that triterpenoids and their saponins can be used as PDE5 inhibitors to inhibit the activity of PDE-5 [52]. In this study, nine major triterpenoids were inferred from PGE using UHPLC-MS, and then these compounds were attached to the active pocket of PDE-5 to form stable complexes, which is similar to the results of Khalid M et al. [1], a study that found that oleanolic acid, a saponin of oleanolic acid, can form stable complexes with the active site of PDE-5. The docking results were also verified in animal experiments, which revealed that SDF and PGE can significantly reduce PDE-5 activity in the penile cavernous tissue. Therefore, it can be inferred that the mechanism of PGE on PRX-induced erectile dysfunction in male mice may be related to the inhibitory effect of triterpenoids on PDE-5.

In addition, saponins have a steroid hormone-like structure, which can be used as a precursor of testosterone synthesis or stimulate testosterone synthesis. It also promotes the synthesis of LH and FSH, and improves the ability of testicular sex hormone synthesis and spermatogenic cell apoptosis [53,54]. In animal models of sexual dysfunction, the levels of T, LH, FSH, and E2 in the serum are also essential indicators for evaluating the strength of sexual ability. When gonadal function declines and sex hormone levels are disordered, it may also lead to different degrees of erectile dysfunction [55]. In this study, the levels of T, LH, FSH, and E2 in male mice after PRX administration were significantly lower than those in the CN group, and the gonadal function of mice was decreased, which is consistent with the results of the study of Yakubu et al. [56]. PRX is an SSRI, which may lead to the disorder of sex hormone secretion by increasing peripheral serotonin levels [57]. However, PGE treatment can improve the disorder of sex hormone secretion in mice and restore sex hormone levels to levels close to those of the CN group, indicating that PGE has the effect of restoring mouse gonadal function and stabilizing hormone levels.

ACP, ALP, and LDH are marker enzymes reflecting the function of testicular Sertoli cells. ACP is mainly present in the cytoplasm of testicular Sertoli cells, which is mainly responsible for the exchange and uptake of nutrients between cells and the clearance of aging or damaged cells. In addition, the abnormality of sperm is also related to the activity of ACP [58]. ALP is highly active in spermatogonia and primary spermatocytes, which is closely related to the division of spermatogenic cells and provides sufficient energy for sperm development [59]. Testicular phosphatase also participates in the synthesis of testicular protein by affecting the secretion of sex hormones and sperm physiology; the changes in the activity of these enzymes will also have a significant effect on the function of sperm [60]. In this study, the administration of PRX resulted in a decrease in phosphatase activity in male mice, which may be related to a decrease in the bioavailability of sex hormones. PGE increased phosphatase activity, indicating that it may enhance testicular function and sex hormone anabolic activity. LDH is a sperm-cell-specific enzyme secreted by testicular spermatogenic cells. It exists in various types of germ cells. Its content and vitality are the highest in sperm, affecting sperm synthesis and release, energy metabolism, and fertilization [61]. PRX significantly reduced the LDH activity in the testis of male mice, thereby reducing sperm synthesis and fertilization ability. After the PGE treatment, the recovery of LDH showed that PGE also promotes spermatogenesis.

In conclusion, PGE is effective in ameliorating PRX-induced sexual dysfunction in male mice, and its mechanism may be related to the rich triterpenoids contained in it, which regulate the sex hormone levels and gonadal organ-related enzyme activities in male mice by mediating the ‘hypothalamus–pituitary–gonadal axis’. In previous studies, Ma et al. [62] found that oleanolic acid has a good protective effect on DNA damage in the testicular tissue of natural aging rats. Zhao et al. [63] also found that oleanolic acid can reduce DNA damage and the apoptosis of germ cells through the inactivation of the NF-κB, p53, and p38 signaling pathways in natural aging rat models, thus effectively restoring testicular function. The testis, as the main organ for testosterone synthesis and secretion, influences male libido, erectile function, and ejaculatory function [64]. It can be concluded that triterpenoid sapogenin (oleanolic acid) has a greater potential in improving male sexual function. In contrast, oleanolic-acid-type saponins have better water solubility and bioavailability than triterpenoid sapogenin (oleanolic acid), which may have better effects than oleanolic acid [65,66]. This seems to have been found in the study by Zhao [67] et al., in which oleanolic acid liposomes improved testicular histological structure and increased testosterone content in a cisplatin-induced mouse model of reproductive disorders. This phenomenon also seems to be found in this study. In this work, UPLC-MS detected nine triterpenoids with a high content from PGE, mainly oleanolic acid saponins. After taking PRX, the gonad function of male mice decreased, mainly manifested as decreased sex hormone levels, testicular tissue structure damage, testicular marker enzyme activity, and sperm quality, which is similar to the results of EL-Gaafarawi et al. [68] Saikia Q [8], and Yakubu et al. [61]. However, after the PGE treatment, the sex hormone levels and testicular tissue structure of mice were effectively improved, which may be related to the oleanolic acid saponins contained in it, which is similar to the results reported in previous studies. In addition, PGE may also play an important role by mediating the NO/cGMP erectile signaling pathway. Mainly, PGE increased the NO content, cGMP content, and NOS activity and decreased PDE-5 activity in the penile tissue of male mice after PRX administration. The results of computer molecular docking also show that triterpenoids in PGE may inhibit PDE-5 activity as PDE-5 inhibitors. Therefore, it is concluded that PGE has an ameliorative effect on PRX-induced sexual dysfunction in male mice. PGE may play a role through multiple pathways, but its exact mechanism needs further investigation. This study provides a new scheme for the future study of the mechanism of PRX-induced sexual dysfunction and also provides a theoretical basis for the development and utilization of *Pfaffia glomerata* and the development of functional foods to improve sexual dysfunction.

## Figures and Tables

**Table 1:** UPLC-MS analysis of the chemical constituents of PGE.

Number	t_R_/min	[M-H]^−^	[M+COOH]^−^	Formula	Fragmentation Ions	Inferred Compounds	Reference
1	0.82	479.3025		C_27_H_44_O_7_	319.1981; 159.1127	20-Hydroxyecdysone	#
2	4.17		1080.3845	C_47_H_70_O_23_S	955.4741; 793.4250; 631.3748	Sulfachyranthoside B	[39]
3	4.45	777.3987		C_41_H_62_O_14_	631.3800; 455.3551	3-O-β-D-Glc-(1→2)-[[2-carboxy-1-(carboxymethyl)-2-hydroxyethyl-(1→3)]-β-D-GluA oleanolic acid	[40]
4	4.72	925.4672		C_46_H_70_O_19_	793.4254; 631.3765; 455.3594	Achyranthoside E	[40]
5	4.82	793.4303		C_42_H_66_O_14_	631.3814; 569.3831; 455.3549	Chikusetsusaponin IVA	#
6	5.16	631.3459		C_35_H_52_O_10_	455.3197; 384.9140; 146.9766	Pfaffiaglycoside B	[41]
7	5.71	793.4293		C_42_H_66_O_14_	631.3812; 569.3831; 455.3578	Zingibroside R1	[40]
8	7.08	955.4397		C_48_H_76_O_19_	793.4292; 631.3809; 569.3834; 455.3556	Achyranthoside C	[41]
9	8.04	631.3823		C_36_H_56_O_9_	455.3552; 146.9765	Calenduloside E	[42]

“#” indicates that it was compared with the standard.

**Table 2:** Effects of PGE on the organ coefficient (%).

Item	Heart	Thymus	Lien	Liver	Ren	Penis	Testis
CN	0.546 ± 0.031	0.081 ± 0.026	0.31 ± 0.017	4.442 ± 0.250	1.535 ± 0.125	0.126 ± 0.008	0.769 ± 0.056
PRX	0.544 ± 0.035	0.081 ± 0.013	0.307 ± 0.065	4.267 ± 0.350	1.512 ± 0.076	0.120 ± 0.005	0.677 ± 0.042 **
PRX + SDF	0.565 ± 0.034	0.081 ± 0.024	0.304 ± 0.033	4.441 ± 0.239	1.515 ± 0.071	0.124 ± 0.011	0.738 ± 0.042 #
PRX + PGE-L	0.536 ± 0.033	0.081 ± 0.024	0.302 ± 0.044	4.314 ± 0.65	1.530 ± 0.035	0.123 ± 0.011	0.737 ± 0.056 #
PRX + PGE-M	0.555 ± 0.038	0.086 ± 0014	0.303 ± 0.018	4.445 ± 0.295	1.536 ± 0.075	0.125 ± 0.009	0.760 ± 0.051 ##
PRX + PGE-H	0.557 ± 0.043	0.082 ± 0.017	0.302 ± 0.031	4.361 ± 0.493	1.528 ± 0.059	0.124 ± 0.013	0.757 ± 0.046 ##

**Note:** The data are expressed as mean ± SD, *n* = 8. Compared with the CN group, ** indicates very significant difference (*p* < 0.01). Compared with the PRX group, # means a significant difference (*p* < 0.05), ## means very significant difference (*p* < 0.01).

**Table 3:** Binding energy score and interactions of the PDE-5 complexes with sildenafil and different compounds in PGE.

Ligands	Binding Energy Score (kcal/mol)	H-Bond Interaction	Interactions
Sildenafil	−9.0	GLN A:817, ARG A:667, ASN A:662	HIS A:613, ASN A:661, LEU A:725, ILE A:824, LEU A:804, ILE A:813, MET A:816, VAI A:782, PHE A:786; THR A:723, LEU A:765, ASP A:764, PHE A:820
20-Hydroxyecdysone	−8.3	GLN A:817, ASP A:764	ILE A:813, ALA A:783, ALA A:779, MET A:816, LEU A:804, LEU A:725, ILE A:824, ASN A:662, ASN A:661, HIS A:617, TYR A:612, LEU A:765
Sulfachyranthoside B	−8.5	GLN A:666, SER A:663	TYR A:664, THR A:802, ASN A:662, LEU A:804, LEU A:725, ILE A:824, PHE A:820, MET A:816, ALA A:823, GLY A:819, SER A:815
Achyranthoside E	−9.1	GLN A:817, SER A:815	GLY A:819, MET A:816, ILE A:665, ILE A:824, PHE A:820, ILE A:813, LEU A:804, PHE A:786, LEU A:765, VAL A:782, GLN A:775, ALA A:767, ILE A:778, ILE A:768, ALA A:779 TYR A:612 HIS A:613, SER A:663 ASP A:803, LYS A:812
Chikusetsusaponin IVA	−8.6	ASN A:662, GLN A:666	LEU A:725, MET A:816, PHE A:820, ILE A:665, TYR A:664, ALA A:823, SER A:663, ILE A:824, GLY A:819, LEU A:804, ARG A:667, ASN A:661, PHE A:786
Pfaffiaglycoside B	−10.3	HIS A:613	ALA A:823; GLY A:819, ILE A:665, ILE A:824, PHE A:820, PHE A:786; LEU A:765, VAL A:782, TYR A:612, THR A:723, ASP A:764, ASP A:724, GLU A:682, HIS A:657, ASN A:661, LEU A:725, LEU A:804, MET A:816
Zingibroside R1	−10.7	VAL A:660, ARH A:667, ASN A:662, HIS A:613, GLU A:682	ILE A:689, SER A:679, MET A:681, ASP A:764, THR A:723, ASP A:724, ILE A:665, SER A:663, LEU A:725, MET A:816, ALA A:824, LEU A:804, PHE A:820, VAL A:782, PHE A:786, ASN A:661
Achyranthoside C	−8.8	GLN A:817, TYR A:612, ALA A:823	GLY A:819, ASN A:662, LEU A:725, SER A:663, ASN A:661, PHE A:786, VAL A:782, GLN A:775, ILE A:786, ALA A:779, ALA A:767, HIS A:617, LEU A:765, LEU A:804, PHE A:820, MET A:816, ASP A:764, ILE A:665, ILE A:824, GLN A:666, ILE A:729
Calenduloside E	−10.0	ASN A:662, HIS A:613	THR A:723, GLU A:682, ASP A:764, ASP A:724, ASN A:662, PHE A:820, LEU A:725, SER A:663, MET A:816, LEU A:804, ALA A:823, ILE A:824, ILE A:665, PHE A:786, VAL 782, LEU A:765, TYR A:612

## Data Availability

Data are available upon request.
